# Coupling multifunction drones with AI in the fight against the coronavirus pandemic

**DOI:** 10.1007/s00607-021-01022-9

**Published:** 2021-11-09

**Authors:** Faris A. Almalki, Abdullah A. Alotaibi, Marios C. Angelides

**Affiliations:** 1 Department of Computer Engineering, College of Computers and Information Technology, Taif University, Taif, Kingdom of Saudi Arabia; 2 Department of Science and Technology, Raniah College, Taif University, Taif, Kingdom of Saudi Arabia; 3 Brunel Design School, College of Engineering, Design and Physical Sciences, Brunel University London, Uxbridge, UK

**Keywords:** Drones, Machine learning, Face mask detection, Internet of everything, COVID-19, 68T07, 68M11

## Abstract

When COVID-19 was declared as a pandemic by the World Health Organization on 11 March 2020, national governments and health authorities across the world begun considering different preventive measures to fight against the coronavirus outbreak. Researchers and tech companies worldwide have been striving to utilize advanced technologies to aid in the fight against the Covid-19 outbreak. This paper aims to couple multifunction drone with AI to deliver wireless services that will help the fight against the Coronavirus pandemic. The proposed drone-eye-system with its thermal imaging cameras and an AI framework utilizes a Convolutional Neural Network (CNN) with its Modified Artificial Neural Network (MANN) for face mask detection of people wearing masks in public. The system can perform basic diagnostic functions such as elevated body temperatures for helping minimize the risk of spreading the infection through close contact. The AI framework evolve an optimized elevation angle $\uptheta$ and altitude ${\mathrm{h}}_{\mathrm{t}}$ to enhance wireless connectivity between a drone and a ground station, which in turn leads to better throughput and power consumption. The proposed framework has been developed using the MATLAB toolbox and shows promising results with an accuracy of face mask detection of 82.63%, with an F1-score of 0.98, and an enhanced by 10% link budget parameters.

## Introduction

**Fig. 1:**
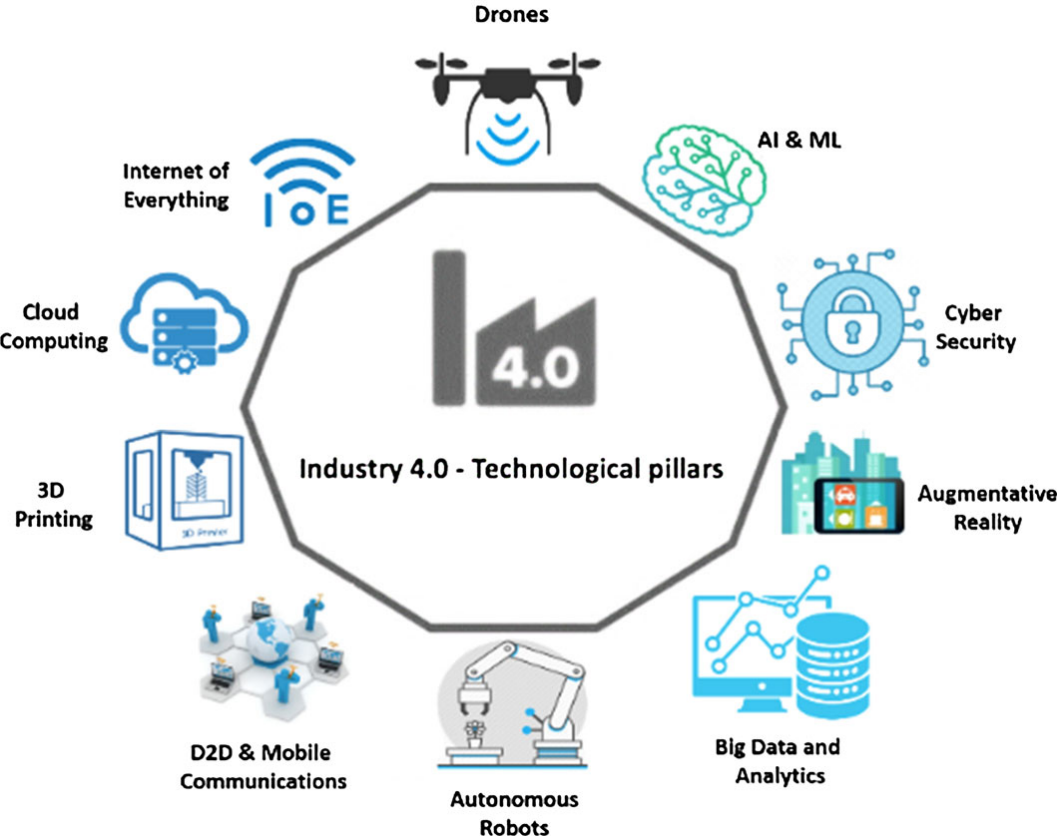
Technological pillars of the 4th industrial revolution [3]

COVID-19 common symptoms may include a severe acute respiratory syndrome, high fever, cough, and shortness of breath. According to the WHO the disease has struck every country across the world resulting in millions of confirmed cases and over a million of confirmed deaths [1]. Authorities worldwide have considered different preventive measures in the fight against the Coronavirus pandemic, such as hand washing, social-distancing, self-isolation, travel restrictions, curfews, and stay-at-home orders. Researchers and tech companies across the globe have joined the race to develop advanced technologies to help the fight against the pandemic. Most of technologies that have evolved relate to the Fourth Industrial Revolution (4IR) and these include Drones, Robotics, Artificial intelligent (AI), Biotechnology, Internet of Everything (IoE), to name a few. Figure 1 shows a more comprehensive list [2, 3].

The rapid advancement of the 4IR has opened doors to limitless possibilities through breakthroughs of emerging technologies in the field of drone technology in partnership with creative digitization. Drone technology provides a cost-effective infrastructure for providing broadband services to fixed and mobile users, like near-space-satellites but without the issues that arise by the distance, high cost or complexity downsides factors. Connectivity, network flexibility, re-configurability, and rapid deployment are advantages of drone technology in providing seamless services over heterogeneous networks at temporary events. A wide range of applications have been taking advantage of drones and IoE for improving smart city infrastructures [4–8].

AI has been serving several crucial roles in the fight against COVID-19 across many areas including, but not limited to, outbreak detection, vaccine development, thermal screening, face mask recognition, and CT scan analysis. An Internet of Medical Things (IoMT) platform that uses machine learning on various devices and mobile applications would be useful in tracking and preventing COVID-19. Such a platform would enable ubiquitous retrieval of real-time patient information without much contact between patients and healthcare workers. UAVs may support such logistics and pave the way in revolutionizing the fight against the Covid-19 outbreak by acting as relays between ground devices and the cloud. This would be particularly useful in remote areas where it may not be economically viable or even possible to achieve within existing infrastructures. UAV-enabled remote sensing of, e.g., elevated temperatures and face-mask recognition would be added value with this technology [1, 9–13].

Facial recognition is a significant approach in empowering security and enabling touchless biometrics for a broad range of purposes. This type of biometric technology brings seamless integration and automation along with fast processing. The COVID-19 pandemic has added additional complexity with the wearing of protective face masks to limit the spread of Coronavirus. Thus, the facial recognition process has become more difficult as the number of facial features available to the process is significantly reduced. The process encounters additional complexities when in addition to face covers, sunglasses, scarf, low hat are worn or when partially facing toward the camera [14–16]. All these scenarios would affect the performance of facial recognition algorithms. Thus, turning to AI to utilize its underlying capabilities of learning, classification, and recognition would be a logical approach.

The overall aim of this paper is to integrate AI into a multifunction drone along with thermal imaging cameras and deploy this drone-eye-system in the fight against the pandemic. The modified drone implements an AI framework for face mask detection and thermal sensing functions such as elevated body temperatures and uses elevation angle and altitude to improve wireless connectivity between the drone and ground stations, for increased throughput and reduced power consumption.

The rest of this paper is organized as follows: section II presents our related research review on drones that are coupled with AI technology and concludes with a presentation of key research gaps, section III presents the AI framework implementation which is the key contribution of our research work, section IV validates our simulation results, and concludes with a complexity analysis and discussion of challenging limitations, and section V concludes with a discussion focused on future perspectives.

## Related research review

This section, firstly, presents a representative sample of the related research works that have been reviewed, compiles a summary of the review in a windup table and then concludes by highlighting research gaps and our own research motivations. We have used the following criteria to source and review related research works that couple UAVs with AI or ML: (a) UAV platform type, (b) standalone topology, (c) propagation model type, i.e., empirical, or deterministic, (d) aerial imaging, i.e., RGB or thermal camera, (e) AI or facial recognition framework, and (f) low altitude missions.

UAVs including drones have a promising potential to revolutionize the future of IoE by working as wireless aerial relays to improve connectivity to ground networks and deliver various wireless communication services. Such a wireless connectivity can be achieved via propagation models which fall into two types as reported in literature: Free space models (e.g. Air-To-Ground (ATG)) [17–21] and Empirical propagation models (e.g. Okumura-Hata, and COST-231) [22–25]. The free-space propagation type focus is a closed-form formula which includes line-of-sight (LoS) and non-line-of-sight (NLoS), and crucially a UAV’s elevation angle. These parameters show a clear tendency towards a wide coverage footprint and a reasonable shadowing effect. The empirical propagation type focus is a pre-defined set of constants and constraints for different geographies. These models can deliver a good level of accuracy, despite some limitations resulting from their limited antenna heights and short transmission coverage. Both types of propagation models feature advantages as well as shortcomings in relation to aspects that affect the performance of any propagation model, e.g., a UAV’s altitude, elevation angle, antenna specifications, and power consumption. Thus, optimization is always required to achieve a wider coverage, moderate power consumption, and better LoS connectivity [3, 17].

Researchers in [26, 27] highlight recent advancements and trends with UAVs for 5G communications. The cutting-edge technologies that is associated with 5G may enable many wireless applications such as wireless sensor networks. Taking advantage of the higher frequency bands of 5G wireless networks, using UAV at 28 and 60 GHz offers much promise for high-capacity wireless communications for such aerial platforms. And yet, our research review informs that the synergy between IoE and UAVs remains an untapped area, which is offering motivation to researchers to pursue further work. Work in [28] presents a NN self-healing model to optimize a UAV’s positioning likelihood for maximizing throughput, coverage, and maximum UE in a 5G network. Results show that the performance of the neural model is an efficient approach based on the upper and lower values of the UAVs’ positioning likelihood along with achieving energy consumption efficiency.

One of UAV’s capability is related to aerial imaging and sensing using different types of equipment, cameras and sensors, for carrying out intelligence, surveillance, and reconnaissance tasks [29]. The complexity in design and fabrication, as well as the challenge of transmitting high quality videos and images to the ground station calls for revisiting antenna design and channel modelling. Researchers in [30] emphasize the dynamic interactions between sensing, coordination, and communication using drones. They also argue that the trade-off between camera quality against their weight is an open issue. Researchers in [31] introduce an innovative approach in which a UAV network uses fog computing to support a natural disaster management system. The proposed work promises flexibility, mobility, and fast deployment features to support IoE systems in smart cities.

The review in [30] highlights open research issues related to drone applications for aerial monitoring, and disaster search and rescue. Reliable communication links between a drone and terrestrial users and/or things is one of the greatest challenges. Where coordination of a drone includes elevation angles and altitudes in addition to the effect of weather conditions to on-board sensors and cameras, the effect on resolution and throughput is significant. The authors argue that on-line decision making for optimization would improve drone’s functionality. The work in [32] highlights some common issues that relate to drone face surveillance, including the effect of motion, differences in pose, illumination, a drone’s altitude, and a camera’s resolution. These effects have a negative relationship with distance between the drone and ground subjects. The paper presents reasonable results using the DroneSURF drone dataset for active and passive surveillance although facial recognition was not significantly effective during evenings due to low source illumination.

Researchers in [33] propose a drone-based smart healthcare system for COVID-19 monitoring, sanitization, social distancing, and data analysis using real-time and simulation-based scenarios. Their framework uses wearable sensors to record the observations in Body Area Networks (BANs) using a thermal camera on board the drone. Results show that a large area can be covered for sanitization, thermal image collection, and patient identification within 2 km and approximately 10 min. In [34] the authors show an implementation of a drone with thermal camera for automatic detection, classification and tracking of objects on the Ocean’s surface. These operations are calculated mathematically using a kernel approximating a Gaussian distribution and Kalman Filter. Although reasonable results are reported, the on-board GPS and Attitude-Heading Reference System (AHRS) are areas suggested for improving the tracking performance.

Authors in [35] present a fast and mobile inter-spectral image registration for real-time monitoring using multiple small-scale drones. Images captured by low-altitude UAVs reveal benefits in surveillance and human detection. However, some challenges are reported such as overlap, scale, rotation, and point of view. Therefore, altitude optimization is recommended for improving results. [36] uses drones with thermal cameras and GPS integrated with Google maps for human detection and geolocation for search and rescue in disaster zones. Experimental results reveal a good level of accuracy in detection by the thermal sensors at an altitude of 12 m and within a radius of 10 m. Researchers in [37] suggest the use of aerial drones with a thermal camera and GPS onboard during foggy weather conditions. They propose road scanning during a fog ahead of police and/or ambulances to reduce the possibility of accidents and assist with their safe and fast navigation to their destination. The altitude and speed of a drone will vary depending on the severity of the weather conditions.

The work in [38] aims to map physical and biological characteristics of unique habitats safely and accurately at an altitude of 20 m using a thermal FLIR Tau sensor with a GPS device attached to a quadcopter drone. Experimental results reveal operational flexibility alongside the high spatial resolutions obtained. It is argued that energy efficiency is a challenge that could be improved by channel optimization. The authors in [39] use a drone that is equipped with thermal camera and a GPS device to calculate the efficiency of solar panels. The aerial thermographic inspection is a useful method in recognizing critical problems in a photovoltaic power plant, in addition to estimating the payback period of maintenance interventions or substitutions.

The work in [40] propose a drone combined with a thermal far-infrared camera for detecting sinkholes from a height of 50 m using a convolutional neural network (CNN) and based on a Boosted Random Forest (BRF) and pattern classification techniques. The simulation results produce reasonable sinkhole detection results. However, the thermal energy of a sinkhole differs significantly, thus detection performance varies. Authors in [41] propose a drone dataset for humans in need of help via action recognition during search and rescue missions. The proposed model uses a CNN based classifier, then non-maximum suppression (NMS) to overcome multiple detections of the same object in an image.

Authors in [42] present an autonomous drone for person identification via taking videos/photos of a target location. In [43] a CNN is introduced to enhance performance of UAV that may act as an artificial eyesight for search and rescue operations for shipwrecked people. The results show that this method offers time efficiency and accuracy. [44] proposes a Convolutional Support Vector Machine (CSVM) network for object detection in UAV imagery. Despite the promising capability of the proposed CSVM network, the configuration reveals high computation time. The paper concludes that recent CNNs could handle multiclass classification issues and thereby produce enhance results. Authors in [45] provide a review on deep learning approaches to applications that include UAVs. CNN, Deep Belief Networks, (DBNs), Deep *Q*-Network (DQN), Deterministic Policy Gradient (DDPG) are approaches that have been considered and reveal satisfactory results.

Researchers in [46] evaluate many factors that affect facial detection and recognition performance from a drone point of view. The empirical study confirms that drones are capable of facial recognition but with some challenges such as distance, angle, and angles of depression. A drone enabled with a camera and face recognition software for human tracking and public safety surveillance is introduced in [47]. In this work, a detected face is color-coded in a square; white for no match, Green for VIP, and Red for blacklisted/missing person. In the event of an escape attempt, the drone can pop-up an alert and give the location coordinates to the pursuing authorities. Results confirm that the proposed framework can reduce human effort, thereby making missions efficient and accurate.

[48] uses a drone with a facial recognition prototype for the purpose of biometric identification, military operations, and surveillance. Results show that despite face detection, there is a need for additional features to become a smarter automated drone. The author in [49] highlight their drone’s efficiency with biometric facial recognition to fight against terrorism. The article concludes that some vital factors should be carefully considered to achieve optimal communication between user and aerial vehicle, battery life, on-board cameras, and AI capabilities.

In [50, 51] a small-size drone equipped with an RGB camera is used in tracking a walking person and robotically capturing a frontal photo of the target from an altitude of 3-4 m. The proposed model uses a deep neural network YOLOv3, Locality-constrained Linear Coding (LLC), and Multi-task Cascaded Convolutional Neural Networks (MTCNN) for person detection, target person matching, and face detection respectively. The model uses a 4G LTE communication module and GPS device to communicate and geolocate the drone. Experimental results verify its effectiveness and practicability.

Table 1 reports the results of a comparative analysis between approaches presented in this section. It helps identify gaps and highlight unresolved issues which are exploited in the remainder of this section.

**Table 1:** Related review windup and comparative analysis between existing approaches

Authors	Optimized Propagation Model	Optimized Elevation Angle ($$\theta )$$	Optimized Altitude ($${h}_{t})$$	Thermal Camera	Aerial Imaging	Facial Recognition	Global Position System (GPS)	Artificial Intelligence (AI)
[4–7]	√	√	√	x	x	x	x	√
[22–25]	√	√	√	x	x	x	x	√
[28]	x	x	x	x	x	x	x	√
[33]	x	x	x	√	√	x	√	x
[34]	x	x	x	√	√	x	x	x
[35]	x	x	x	√	√	x	x	x
[36–39]	x	x	x	√	√	x	√	x
[40]	x	x	x	√	√	x	x	√
[41]	x	x	x	x	√	√	x	√
[42]	x	x	x	x	√	√	√	x
[43, 44]	x	x	x	x	√	x	x	√
[45]	x	x	x	x	√	x	√	√
[47]	x	x	x	√	√	√	x	x
[48]	x	x	x	x	√	√	√	x
[49, 50]	x	x	x	x	√	√	√	√

The research review has revealed a few challenges that need to be addressed to enhance the deployment of a multifunction drone with AI in the fight against the Coronavirus pandemic. These include:

- Lack of an optimized channel model for aerial imaging
- Need for a trade-off between ${\mathrm{h}}_{\mathrm{t}}$ against image resolution and transmission throughput
- Lack of optimized $\uptheta$ and ${\mathrm{h}}_{\mathrm{t}}$ for aerial imaging
- Need for trade-off between throughput and power consumption
- Lack of consideration of thermal imaging for face mask detection from a drone’s point of view
- Lack of consideration of an integrated payload consisting of a transceiver payload, GPS, and thermal imaging camera
- Lack of consideration of coupling AI with partial facial detection from a drone point of view
  After careful consideration of the above shortcomings, we propose to exploit the use of an aerial drone system in pursue of the following research objectives:
- Channel optimization by tuning $\uptheta$ and ${\mathrm{h}}_{\mathrm{t}}$ to enhance wireless connectivity, throughput, and power consumption
- Design of an AI framework for partial face mask detection
- Deployment of thermal imaging camera to sense body temperatures a main symptom of Covid-19
- Deployment of a transceiver payload with GPS for geolocation tracking of persons with elevated body temperatures, who violate curfews or stay-at-home orders

## Proposed framework implementation

**Fig. 2:**
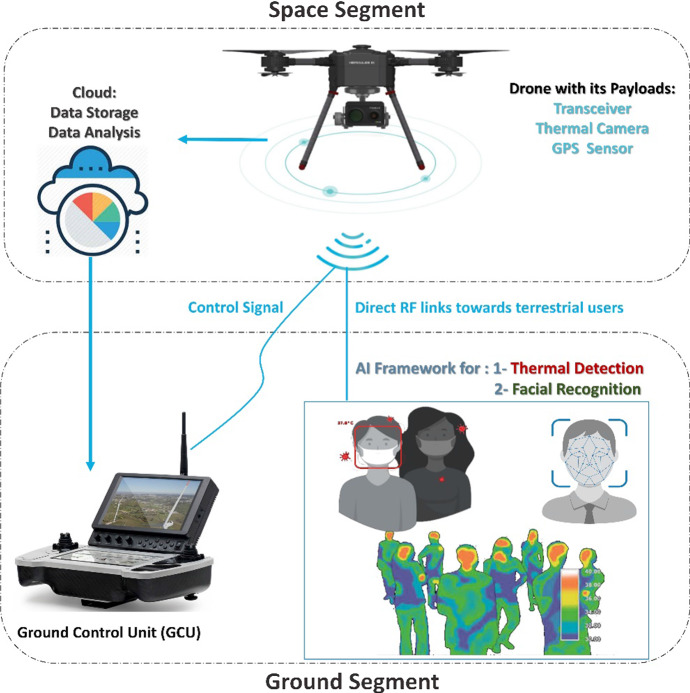
Conceptual architecture of the proposed model AI framework

**Fig. 3:**
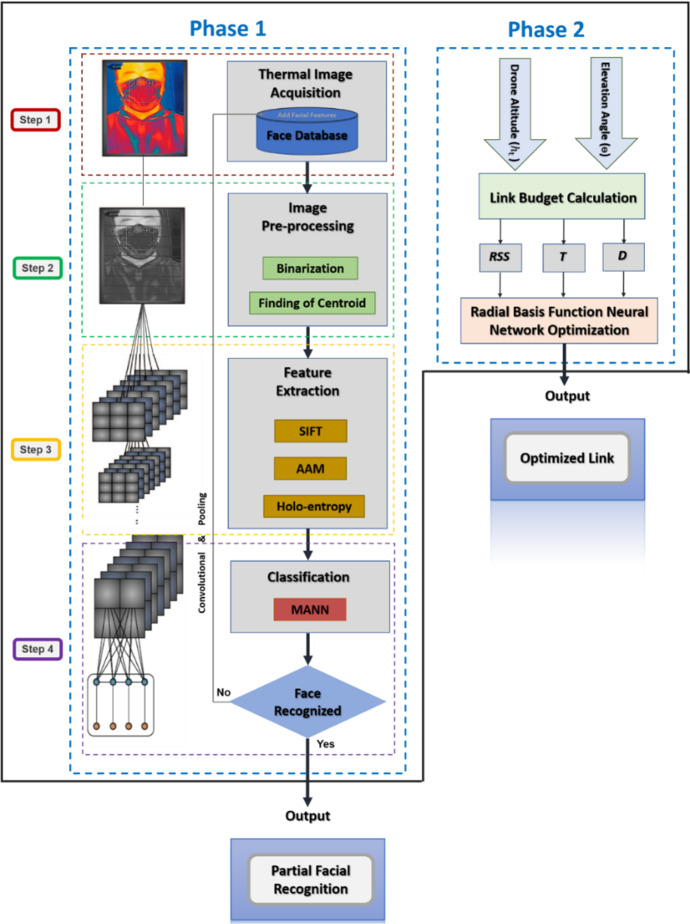
The proposed AI framework

This section presents the physical architecture underpinning the implemented AI framework which is shown on Fig. 2, and then discusses the AI framework which is illustrated on Fig. 3. The principal aim of the work is to integrate AI in a multifunction drone that helps deliver a wireless service, from basic diagnostic functions that will help with minimizing the risk of spreading infection to face mask detection to tracking individuals [51–53].

The proposed conceptual model on Fig. 2 shows a drone-eye-view along with its payloads in the space segment with the ground segment containing a Ground Control Unit (GCU) which executes the AI Framework with data received from the Drone. The architecture serves a list of functions: Firstly, face mask detection to identify a person who is wearing a mask and may be exhibiting anti-social behaviour or violating curfews and stay-at-home orders. Secondly, thermal imaging to sense elevated body temperatures and geolocation tracking of persons with elevated body temperatures to minimize the spread of infection. Thirdly, geolocation tracking of persons who exhibit anti-social behaviour or violate curfews and stay-at-home orders. Fourthly, wireless communication between the drone and (a) the GCU using a control signal, and (b) terrestrial users using direct Radio Frequency (RF) links.

Drones are regarded as a fast and low-cost alternative to setting up an eye-in-the-sky because they feature many of the satellite advantages but without the distance penalty. Furthermore, terrestrial receivers may offer a higher signal quality as the RF links may be at LoS, and, thus, experience less propagation delay. The prototype uses a DJI Quadcopter with various onboard payloads, a thermal camera, and a transceiver. The transceiver collects data using the thermal camera and then transmits the data to the cloud for storage and further analysis. The thermal camera is loaded with a GPS sensor, so when a person that has been identified has a higher-than-normal temperature, or is in violation of a stay-at-home order, their geo-location can be retrieved, and their movement tracked for containment.

Figure 3 illustrates the proposed AI framework that carries out its tasks in a two-phase approach. During the first phase, it optimizes the link budget parameters to enhance wireless connectivity between a drone and the GCU by tuning the drone's $\uptheta$ and ${\mathrm{h}}_{\mathrm{t}}$ using the Radial Basis Function (RBF) Neural Network Tool. During the second phase, the framework carries out face mask detection of persons wearing face masks using a Convolutional Neural Network (CNN) with its Modified Artificial Neural Network (MANN) [51–53].

### Phase 1: optimizing link budget parameters

This phase aims to establish enhanced wireless communication links between a drone and its ground station by optimizing link budget parameters. This can be done by tuning the input parameters of drone altitude $({\mathrm{h}}_{\mathrm{t}})$, and elevation angle $\left(\theta \right)$, which reportedly affect wireless connectivity in any space-based communication system [32, 53]. To carry out the optimization, the RBF-NN tool is used as it supports data selection, network creation and training, and network performance evaluation using MSE and regression analysis. The optimization aims at maximizing Received Signal Strength (RSS), throughput (T) and coverage distance (D) whilst minimizing transmission power and consumption.

The framework uses a two-rays propagation model as it offers advantages in relation to altitude, wide coverage range, adaptation with both LoS and NLoS conditions in an urban environment. The two-rays model takes into consideration various elevation angles along with altitudes between a drone and mobile or stationary terrestrial users which in turn leads into enhanced connectivity and greater support for peer-to-peer (P2P) connections [3]. The path loss (PL) of the two-rays model and the link budget parameters can be expressed by Eqs. (1) through to (5).

1 $$ \text{PL} \left[ {\text{dB}} \right] = 40\log (\text{d}) \, {-} \, [10 \, \log \, \text{G}({\text{h}_{\text{t} } })  + 10 \, \log \, \text{G}({\text{h}_{\text{r} } })  + 20 \log \left( {\text{h}_{\text{t} } } \right) + 20 \log \left( {\text{h}_{\text{r} } } \right)] $$

2 $$ {\text{d}} \, \left[ {\text{km}} \right] \, = 2 {\text{E}}_{\text{r}} \left[ {\cos^{ - 1} \left( {\frac{{{\text{E}}_{\text{r}} }}{{\text{E}}_{\text{r}} + {\text{h}}_{\text{t} } }*\cos \left( \theta \right)} \right) - \theta } \right] $$

3 $$ {\text{RSS}} = {\text{P}}_{{\text{t}}} + {\text{G}}\left( {{\text{h}}_{{\text{t}}} } \right){ } + {\text{G}}\left( {{\text{h}}_{{\text{r}}} } \right){ } - {\text{PL}} - {\text{L}} $$

4 $$ {\text{SNIR}} = \frac{{{\text{RSS}}}}{{{\text{N}} + {\text{I}}}} $$

5 $$ {\text{T}} = {\text{B}} \times {\text{log}}\left( {1 + {\text{SNIR}}} \right) $$

where distance ${\text{d}}$ of the propagation model’s range is computed based on $\theta$, $\mathrm{G}\left({\mathrm{h}}_{\mathrm{t}}\right)$ denotes transmitter antenna height gain, $\mathrm{G}\left({\mathrm{\text{h}}}_{\mathrm{r}}\right)$ denotes receiver antenna height gain, ${\mathrm{h}}_{\mathrm{t}}$ denotes drone’s altitude, ${\mathrm{h}}_{\mathrm{r}}$ denotes receiver antenna height, ${\text{E}}_{\text{r}}$ denotes the Earth’s radius at 6378 km, ${\mathrm{P}}_{\mathrm{t}}$ denotes transmitter power, $\mathrm{L}$ denotes connector and cable loss, $\mathrm{N}$ denotes Noise figure, $\mathrm{I}$ denotes Interference, B denotes bandwidth, SINR denotes signal-to-interference-ratio [3].

Before using the RBF-NN tool to optimize the link budget parameters of RSS, T, and D a random representative input sample from the initial predictions are presented to the neural network for training. After training the predicted results are interred as inputs ${(\mathrm{x}}_{1}$, ${\mathrm{x}}_{2}$, ${\mathrm{x}}_{3}$,… ${\mathrm{x}}_{\mathrm{n}}$) to the RBF-NN. At the hidden layer the neurons are activated by the RBF-NN using Gaussian functions and a nonlinear transformation is applied to the input variables. The optimized parameters are obtained at the output layer. The learning rate is accelerated, and the usual local minimum issue is avoided. The layers of RBF-NN can be mathematically expressed by Eqs. (6) through to (11).

6 $$ {\text{X}} = \, [{\text{x}}_{1} ,{\text{ x}}_{2} ,{\text{ x}}_{3} , \ldots .{\text{ x}}_{{\text{n}}} ]^{{\text{T}}} $$

7 $$ {\text{H}} = \, [{\text{h}}_{1} ,{\text{ h}}_{2} ,{\text{ h}}_{3} , \ldots .{\text{ h}}_{{\text{n}}} ]^{{\text{T}}} $$

8 $$ {\text{h}}_{{\text{n}}} = {\text{exp }}\left( { - { }\frac{{{\|\text{X}} - {\text{ C}}_{{\text{n}}}\|^{2} }}{{2{\text{ b}}_{{\text{n}}}^{2} }}{ }} \right)^{ } $$

9 $$ {\text{C}} = \, [{\text{C}}_{1} ,{\text{ C}}_{2} ,{\text{ C}}_{3} , \ldots .{\text{ C}}_{{\text{n}}} ]^{{\text{T}}} $$

10 $$ {\text{B}} = \, [{\text{b}}_{1} ,{\text{ b}}_{2} ,{\text{ b}}_{3} , \ldots .{\text{ b}}_{{\text{n}}} ]^{{\text{T}}} $$

11 $$ {\text{W}} = \, [{\text{w}}_{1} ,{\text{ w}}_{2} ,{\text{ w}}_{3} , \ldots .{\text{ w}}_{{\text{n}}} ]^{{\text{T}}} $$

where X are the predictions used as training input denoted in (6), H is the radial vector of the RBF network denoted in (7)*,* h_n_ is a multivariate Gaussian function denoted in (8), C is the centre vector of the network denoted in (9), B is the radial width vector denoted in (10), and W is the weight vector of the network denoted in (11). These parameters are required to predict the output of the RBF-NN.

At the start of the learning phase the connection weights in ${\mathrm{W}}_{0\mathrm{j}}$ are distributed as follows: RSS = 0.5, D = 0.2, and R = 0.3. The higher weight of RSS sets out channel performance as a top priority during setup. Nevertheless, the weights of ${\mathrm{W}}_{\mathrm{ji}}$ will get updated in the perceptron of the hidden layer as the learning phase proceeds. Equations (12) and (13) represent the network output as a linearly weighted sum of the number of base functions in the hidden layer.

12 $$ {\text{O}} = {\text{F}}_{0} \left({\mathop \sum \limits_{{{\text{j}} = 0}}^{{\text{N}}} {\text{W}}_{{0{\text{j}}}} { }\left({{\text{F}}_{{\text{h}}} \left( {{ }\mathop \sum \limits_{{{\text{i}} = 0}}^{{\text{N}}} {\text{W}}_{{{\text{ji}}}} {\text{h}}_{{\text{n}}} { }} \right){ }} \right){ }} \right) $$

13 $$ {\text{O}} = \left( {{\text{W}}_{{{\text{RSS}}}} + {\text{h}}_{1} {\text{W}}_{1} { }} \right) + { }\left({{\text{ W}}_{2} + {\text{h}}_{2} {\text{ W}}_{2} { }} \right) + { }\left({{\text{ W}}_{3} + {\text{h}}_{3} {\text{ W}}_{3} { }} \right) $$

where, ${\mathrm{F}}_{\mathrm{h}}$ and ${\mathrm{F}}_{0}$ are the activation functions of the neurons in the hidden and output layers respectively. Selecting the most optimal value is fulfilled by applying k-means clustering using a Gaussian mixture distribution as shown in Eqs. (14) through to (17). This is achieved by specifying the beta coefficients $\upbeta$, and setting sigma $\upsigma$ to the mean distance between the cluster centre and other points in the cluster. K-means starts by initializing the centre for the first pattern of the cluster, which includes the optimized values of different parameters at various aerial platform altitudes and elevation angles. During selection of the best optimized value, the network adaptively fine-tunes the free system parameters based on the corrections which minimize the MSE between inputs ${\mathrm{y}}_{\mathrm{i}}$ and the desired output ${\mathrm{d}}_{\mathrm{i}}$, which represents the parameter bounds that are considered will improve channel performance. RBF-NN neurons compete at every iteration until either there are no further centre updates, or the maximum number of iterations is reached [32, 53, 54].

14 $$ {\upsigma } = \frac{1}{{\text{m}}}\mathop \sum \limits_{{{\text{i}} = 1}}^{{\text{m}}} \left| {\left| {{\text{x}}_{{\text{i}}} - {\upmu }} \right|} \right| $$

15 $$ \upbeta = \frac{1}{{2\upsigma^{2} }} $$

16 $$ {\text{p}}\left( {\text{x}} \right) = \mathop \sum \limits_{\text{i} = 1}^{\text{K}} \pi_{\text{k}} {\mathcal{N}}\left( {{\text{x}}|\upbeta \mathop \sum \limits_{\text{i}} } \right) $$

17 $$ {\text{MSE}} = \frac{1}{2}{ }\mathop \sum \limits_{{{\text{j}} = 1}}^{{\text{N}}} ({\text{y}}_{{\text{i}}} - {\text{d}}_{{\text{i}}} { })^{2} $$

where, $\mathrm{m}$ denotes the number of training samples belonging to cluster, ${\mathrm{x}}_{\mathrm{i}}$ denotes the ith training sample in the cluster, $\upmu$ denotes the cluster centroid, $\mathrm{p}(\mathrm{x})$ denotes Gaussian data of K number of distributions, ${\uppi }_{\mathrm{k}}$ denotes the proportion of data generated by the k-th distribution, $\mathcal{N}(\mathrm{x}|\upbeta \sum \limits_{\text{i}})$ denotes the multidimensional Gaussian function with mean vector $\upbeta$ and covariance matrix $( \sum \limits_{\text{i}})$ [32, 33]. The stages above inform the output matrix that may help responsible authorities in making decisions that will ensure safety and security protocols are upheld:

- Recognition of a person through face mask detection or storing of the face features as new data in the database, if a match was negative,
- Elevated body and head temperature in Celsius, and
- Optimized link budget parameters of RSS, T, and D

### Phase 2: face mask detection

This phase is carried out across four steps: Image Acquisition (Step 1), Image Pre-processing (Step 2), Feature Extraction (Step 3) and Classification (Step 4), as shown on Fig. 3.

*Step 1* During the image acquisition step, thermal imaging is continuously acquired with variable expressions for multiple persons regardless of whether protective masks are worn. These are then stored in a database for processing and further use during subsequent steps. During this step, a thermal camera is used to detect radiating heat from a body, usually from the forehead, and this is then used to estimate a body temperature. This is a vital piece of information in the fight against the spread of the pandemic as an elevated temperature is one of coronavirus key symptoms, and early detection may help with minimizing the risk of spreading the infection. The thermal camera may be linked with a GPS sensor, so when a person is identified as having a higher-than-normal temperature, or as violating curfews and stay-at-home orders, their geo-location can be traced, and their movement tracked for the relevant authorities to take the right course of containment action.

*Step 2* During the image pre-processing step, thermal images are converted from colour to grayscale images, binarized, and the face centroid determined. The process of binarization can be mathematically expressed by Eqs. (18) through to (20). Finding the face centroid is expressed by Eqs. (21) and (22).

18 $$ {\text{b}} \, \left( {{\text{i}}, {\text{j}}} \right) =\left\{ \begin{array}{*{20}l} 1 \hfill & {{\text{if}}\, \, \text{g} \, \left( {{\text{i}},{\text{j}}} \right) \, \ge {\text{g}}_{{\text{mean }}} } \hfill \\ 0 \hfill & {{\text{otherwise}}} \hfill \\ \end{array} \right. $$

19 $$ {\text{g}}_{{\text{mean }}} = \frac{{\mathop \sum \limits_{{{\text{i}} = 1}}^{{{\text{row}}}} \mathop \sum \limits_{{{\text{j}} = 1}}^{{{\text{column}}}} {\text{ g }}\left( {{\text{i}},{\text{j}}} \right)}}{{({\text{row }} \times {\text{column})}}} $$

20 $$ {\text{I}} \, = \, (0.298 \times {\text{red}}_{{\text{component}}}) + \, (0.587 \times {\text{green}}_{{\text{component}}} ) + \, (0.114 \times {\text{ blue}}_{{\text{component}}}) $$

21 $$ {\text{X}} = \frac{{\sum {\text{m}}_{{{\text{f}}\left( {\text{x}, \text{y}} \right)^{\text{x}}}}}}{{\sum {\text{m}}_{{{\text{f}}\left( {\text{x}, \text{y}} \right)}} }} $$

22 $$ {\text{Y}} = \frac{{\sum {\text{m}}_{{{\text{f}}\left( {\text{x}, \text{y}} \right)^{\text{y}} { }}} }}{{\sum {\text{m}}_{{{\text{f}}\left( {\text{x}, \text{y}} \right)}} }} $$

where I is grayscale image, ${\mathrm{g}}_{\mathrm{mean}}$ is mean gray value of grayscale image, b (i, j) is binary image where black pixels means background is represented with ‘0’s while white pixels means background is represented with ‘1’s, g (i, j) represent the ${\mathrm{red}}_{\mathrm{component}}$, ${\mathrm{green}}_{\mathrm{component}}$, and ${\mathrm{blue}}_{\mathrm{component}}$ of an RGB color image respectively, (x, y) are the coordinates of a binary image, m is the intensity value that is ${\mathrm{m}}_{\mathrm{f}(\text{x}, \text{y})}$ = ${\mathrm{f}}_{(\text{x}, \text{y})}$ = 0 or 1.

*Step 3* During the feature extraction step, CNN techniques are used to extract features from the grayscale image: Scale Invariant Feature Transform (SIFT), Active Appearance Model (AAM), and Holo entropy. The SIFT descriptor layer detects and describes specific local features of an image such as the orientation and the spatial relationship of the features extracted by the convolutional layer. AAM uses a statistical model to annotate a face shape, coordinates, and appearance to a new image. Holo entropy uses entropy and correlation information to detect outliers and uniqueness on a face image. These features can be mathematically expressed by Eqs. (23) through to (30).

23 $$ \text{L} \, (\text{a}, \, \text{b},\upsigma ) \, = \, \text{G} \, (\text{a}, \, \text{b},\upsigma ) \times \text{I} \, \left( {\text{a}, \, \text{b}} \right) $$

24 $$ \text{G} \left( {\text{a}, \text{b}, \upsigma } \right) = \frac{1}{{2\pi \upsigma^{2} }}\text{e}^{{\frac{{ - \text{a}^{2} + \text{b}^{2} }}{{2\upsigma^{2} }}}} $$

25 $$ \text{D} \left( {\text{a}, \text{b}, \upsigma } \right) = {\text{L}} \left( {\text{a}, \text{b},\text{k} \upsigma } \right) - {\text{L}} \left( {{\text{a}}, {\text{b}}, \upsigma } \right) $$

26 $$ {\text{E}} = \frac{{\partial^{2} { }{\text{D}}^{ - 1} }}{{\partial {\text{a}}^{2} }} - { }\frac{\partial {\text{D}}}{{\partial {\text{a}}}} $$

27 $$ {\text{D}}_{\text{m}} \left( {\text{a},{ } \text{b}} \right) = \sqrt {\begin{array}{*{20}c} {\text{L}\left( {\text{a} + { }1, \text{b}} \right) - {\text{L}}\left( {\text{a} - 1, \text{b}} \right) + (\text{L}\left( {\text{a},{ }1 + \text{b}} \right) - {\text{L}}\left( {\text{a},{ }1 - \text{b}} \right))^{2} } \\ { } \\ \end{array} } $$

28 $$ \uptheta \left( {\text{a}, \text{b}} \right) = \frac{{\tan^{ - 1} \left( {\text{L}\left( {\text{a}, 1 + \text{b}} \right) - \text{L}\left( {\text{a}, 1 - \text{b}} \right)} \right)}}{{\left( {\text{L}\left( {\text{a} + 1, \text{b}} \right) - \text{L}\left( {\text{a}, 1 - \text{b}} \right)} \right)}} $$

29 $$ \text{H}_{\text{D}} \left( \text{V} \right) = \text{H}_{\text{D}} \left( {\text{V}_{1} } \right) + \text{H}_{\text{D}} \left( {\text{V}_{2} } \right) + \text{H}_{\text{D}} \left( {\text{V}_{3} } \right) + \ldots \text{H}_{\text{D}} \left( {\text{V}_{\text{m}} |\text{V}_{\text{m} - 1} , \text{V}_{1} } \right) $$

30 $$ {\text{HL}}_{\text{D}} \left( \text{V} \right) = \mathop \sum \limits_{\text{i} = 1}^{\text{m}} \text{H}_{\text{D}} \left( {\text{V}_{\text{i}} } \right) $$

where L (a, b, $\upsigma$) represents the evaluation search over image scales and orientation of convolution with a variable-scale Gaussian G (a, b, $\upsigma$), I (a, b) is the input image, and $\mathrm{D }\left(\mathrm{a},\mathrm{ b},\upsigma \right)$ represents a Taylor expansion of the scale-space function. Key points are selected based on measures of stability via E, a location of extremum, whose value should be carefully considered against a threshold as one below means poorly localized candidate, whereas above means accurately locating key feature points by discarding indistinct key info points.

*Step 4* During the classification step, MANN categorizes a facial image into recognized or not recognized using a backpropagation algorithm for training purposes and a firefly algorithm for correcting the placement of neuron weights. These can be mathematically expressed by Eqs. (31) through to (37).

31 $$ \text{I} = \upalpha + \mathop \sum \limits_{\text{n} = 0}^{{\text{H}_{\text{u} - 1} }} \text{w}_{\left( \text{n} \right)} \text{F}_{1} \left( \text{n} \right) + \text{w}_{\left( \text{n} \right)} \text{F}_{2} \left( \text{n} \right) + \ldots \text{w}_{\left( \text{n} \right)} \text{F}_{\text{m}} \left( \text{n} \right) $$

32 $$ {\text{fitness}} = \min \mathop \sum \limits_{i = 1}^{n} {\text{MSE}} $$

33 $$ {\text{w}}_{\text{i}}^{{{\text{new}}}} = \text{w}_{\text{i}} + \text{A}_{\text{t}} \left( {\text{w}_{\text{j}} - \text{w}_{\text{i}} } \right) + \upalpha \left( {\updelta - \frac{1}{2}} \right) $$

34 $$ \text{A}_{\text{t}} = \text{A}_{\text{t0}} \text{e}^{{ - \upbeta \text{D}_{\text{ij}}^{2} }} $$

35 $$ {\text{Active}} \left( \text{I} \right) = \frac{1}{{1 + \text{e}^{ - \text{I}} }} $$

36 $$ \text{O} = \frac{1}{2}\mathop \sum \limits_{\text{n} = 0}^{{\text{H}_{{\text{u}} - 1}}} (\text{D}_{\text{n}} - \text{A}_{\text{n}} )^{2} $$

37 $$ {\text{Result}} = \left\{ {\begin{array}{lll} {{\text{Recognized}}, \quad \text{O} < \text{w},} \\ {{\text{Not}}\,{\text{recognized}}, \quad \text{O} \ge \text{w}} \\ \end{array} } \right. $$

where ${\text{F}}_{1}\left(\text{n}\right)$ is input units, ${\text{H}}_{\text{u}}$ is hidden units, ${\text{w}}_{\left(\text{n} \right)}$ is weight which can be updated for each solution of the firefly algorithm. The fitness value of each solution is estimated using mean square error (MSE) which represents the shortlisted value as the current best weights. $\upalpha$ and $\updelta$ represent the uniformly spread values in the range of 0 to 1. ${\text{A}}_{\text{t0}}$, $\upbeta$, and ${\text{D}}_{\text{ij}}$ are pre-set attractiveness, light absorption coefficient, and distance between the ${\text{i}}^{\text{th}}$ and ${\text{j}}^{\text{th}}$ neighbouring solution respectively. $\text{Active} \left(\text{I}\right)$ represents the activation function for the output layer, ${\text{A}}_{\text{n}}$ is actual outputs, ${\text{D}}_{\text{n}}$ is desired outputs, and $\text{O}$ is the output unit. The Error is expected to be at a minimum value to get a well-trained network. This can be done via fixing a threshold value against the output value. If the output is below the threshold, the face image is recognized otherwise, it is not.

## Simulation and validation

This section, firstly, visualises and validates simulation results, and then undertakes a complexity analysis and concludes with a discussion of challenging limitations to any researcher whose work involves the use of drones.

### Visualisation of simulation results

**Fig. 4:**
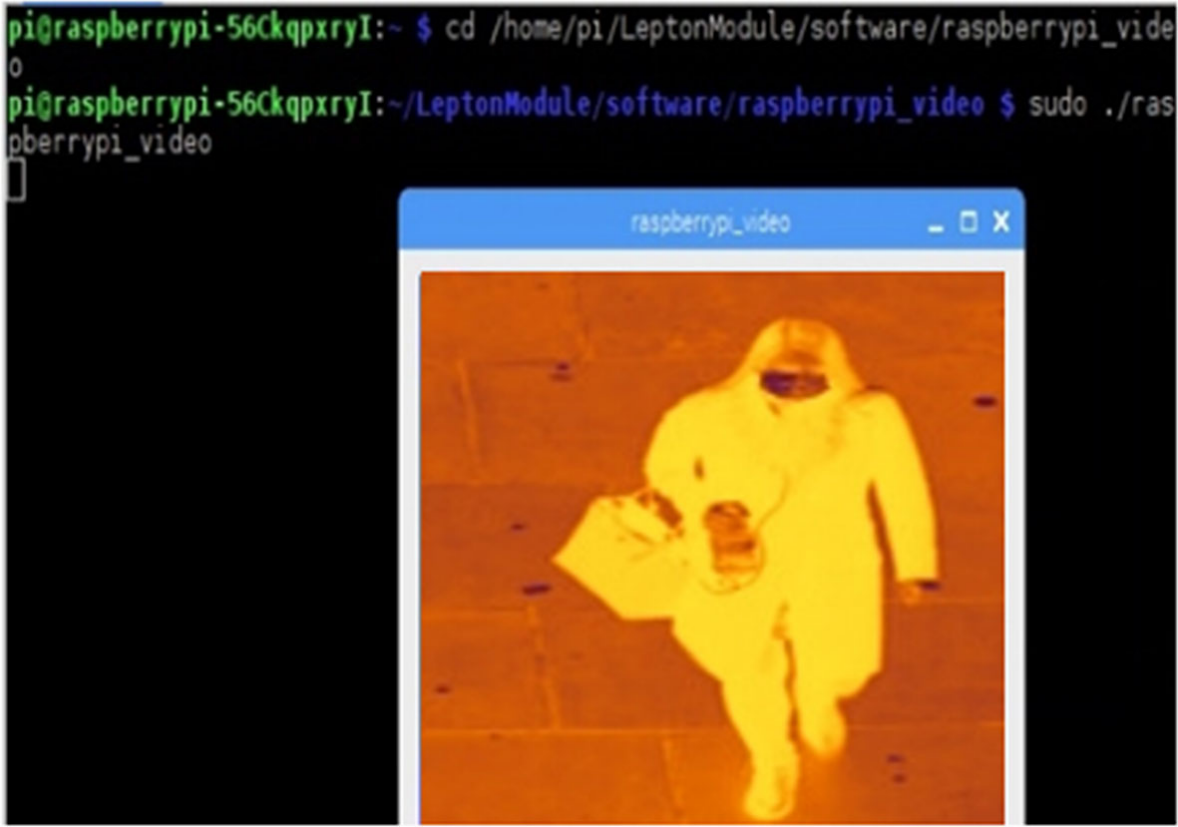
Building a dataset of thermal images

**Fig. 5:**
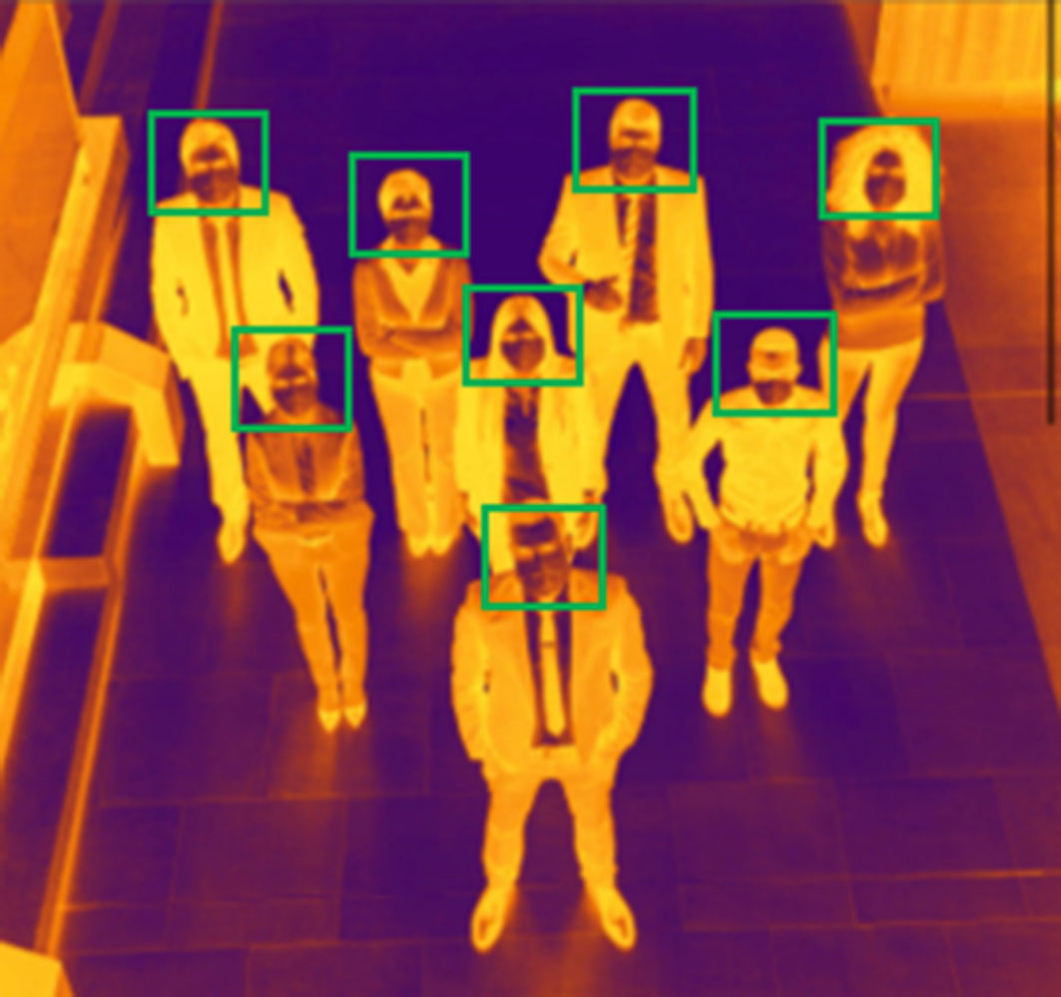
Training dataset of thermal images

**Fig. 6:**
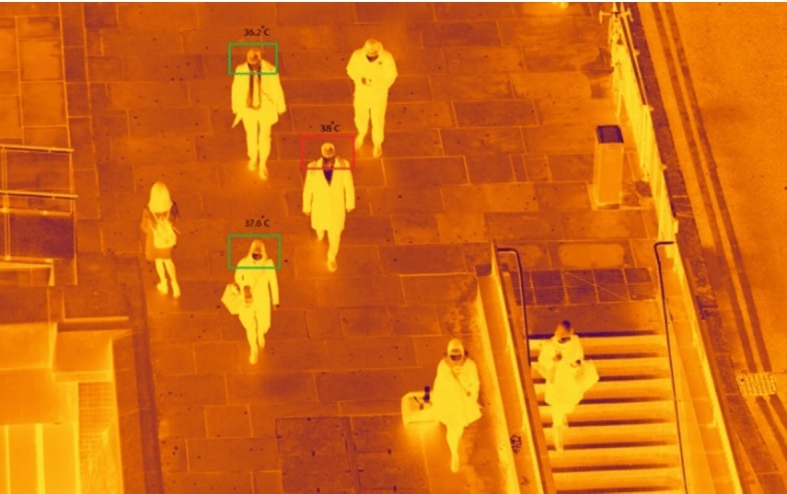
Validation dataset of thermal images with temperature sensing

Our entry point to the simulation is the primary dataset of the thermal imaging obtained by the thermal camera FLIR Lepton, as Fig. 4 shows. This is divided into two subsets: a training set for the neural net as shown on Fig. 5, and a testing and validating set for face mask detection as shown on Fig. 6. Random sets are used for sensing elevated temperature in Celsius as also shown on Fig. 6. When training the neural network, we consider the 5G MIMO antenna specifications provided by the Taoglas Company [55]. We tune the drone's elevation angle and altitude to predict the optimized link budget parameters with $\uptheta$ varied between 5° and 90° and ${\mathrm{h}}_{\mathrm{t}}$ floating between 10 and 100 m above ground. All predictions are obtained by applying Eqs. 1–37 in MATLAB simulation toolboxes.

**Fig. 7:**
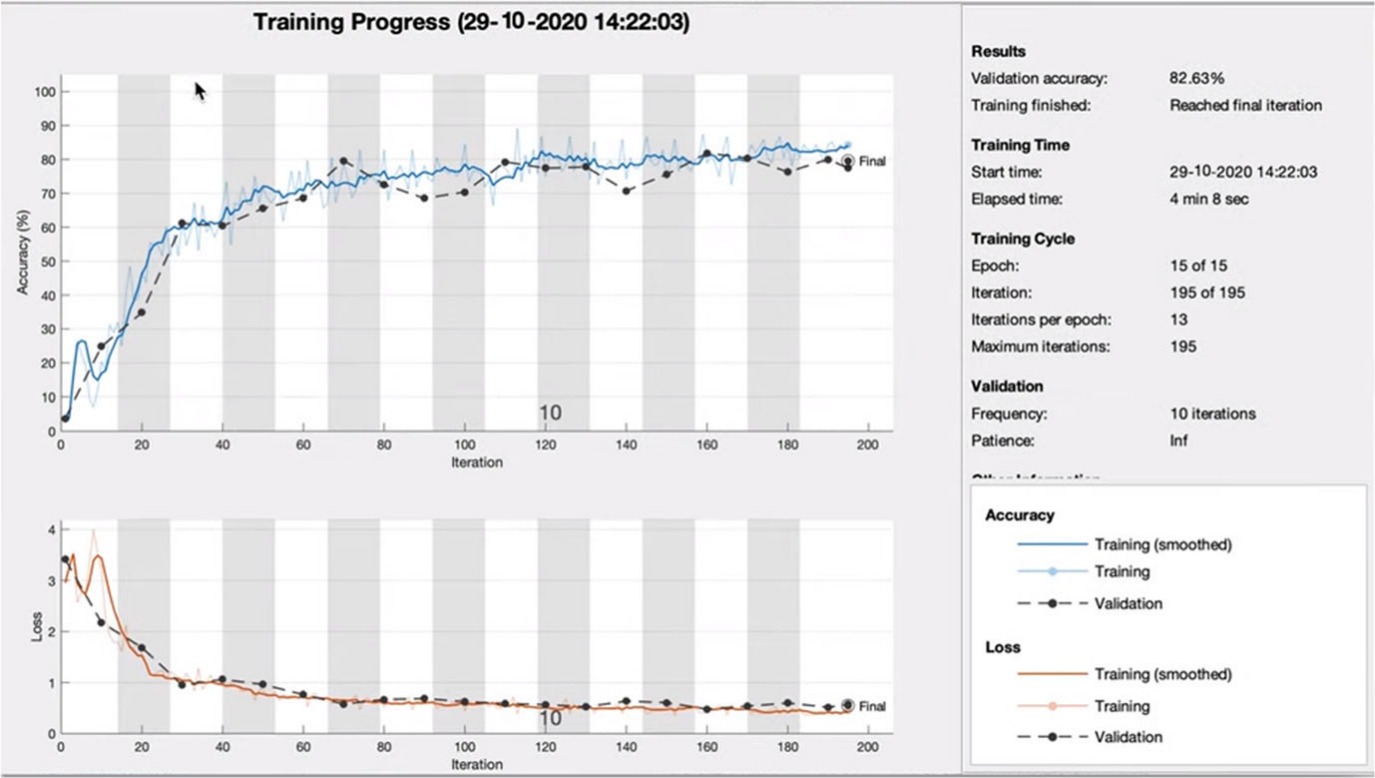
Neural network training accuracy

**Fig. 8:**
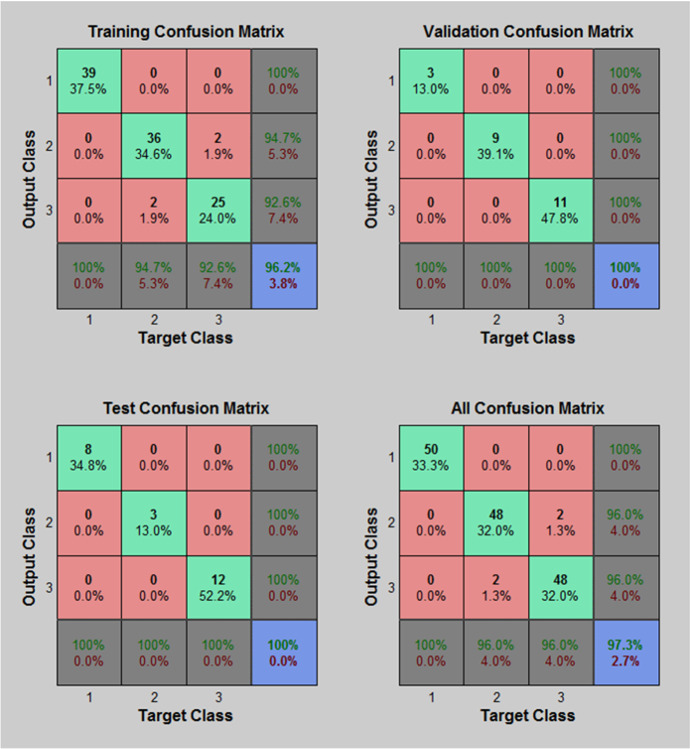
Confusion matrix

Figure 7 shows the neural network training accuracy against loss. This shows an accuracy of 82.63% with face mask detection. A Confusion matrix is used to validate quantitatively the results as shown on Fig. 8. The facial images classified appeared in green squares, whilst those that remain unclassified appear in red squares. The confusion matrix is one of the well-known performance indicators in a classification algorithm. This is done by calculating Sensitivity, Precision, and accuracy out of all positive classes, i.e., how much has been predicted correctly. To give more depth to our validation an F1-score is used to represent the harmonic mean of precision and recall together.

Table 2 shows the confusion matrix performance of the proposed face mask detection using CNN with MANN. The TABLE shows that the confusion matrix confirms that the thermal images that are used in training, testing and validations are properly classified with a high F1-score close to the value of 1. In turn this confirms that the proposed AI framework is working well with a high level of accuracy.

**Table 2:** Confusion matrix of proposed ai framework

Dataset	Sensitivity	Precision	Accuracy	F1-score
Training dataset	0.99	0.54	0.95	0.98
Validation dataset	0.98	0.54	0.94	0.973

**Fig. 9:**
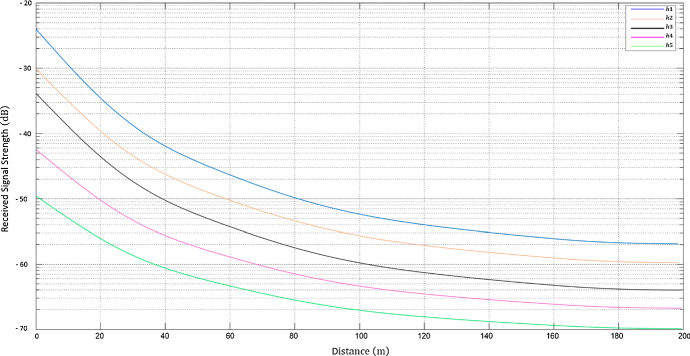
RSS predictions as function of D at different drone altitudes

**Fig. 10:**
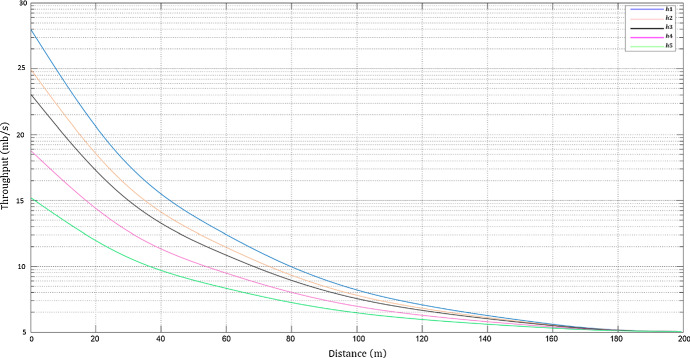
T predictions as function of D at different drone altitudes

**Fig. 11:**
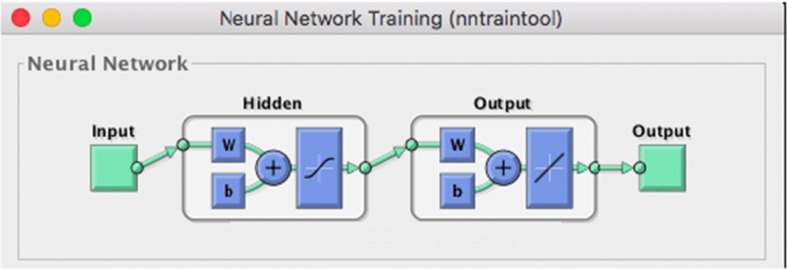
RBF-NN layout in MATLAB

**Fig. 12:**
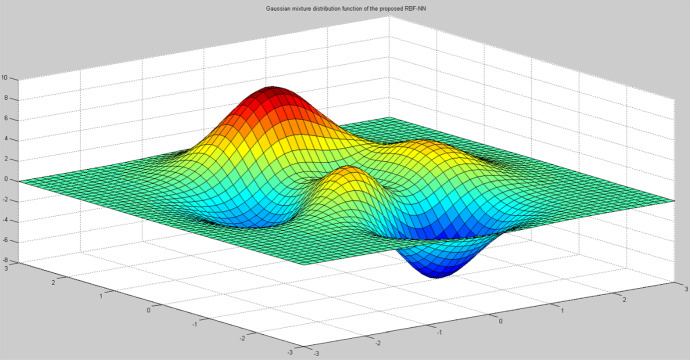
RBF-NN as a Gaussian mixture distribution function

**Fig. 13:**
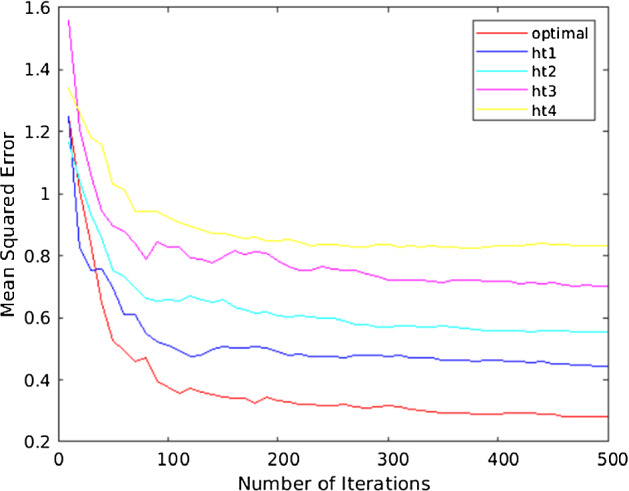
The MSE performance of the proposed RBF-NN in MATLAB

The predicted results on a drone’s altitudes and elevation angles that are used in RBF-NN are shown on Figs. 9 and 10 as RSS and T predictions as function of D respectively, at different drone’s altitudes. Minimizing PL whilst maximizing RSS, T and D at various drone altitudes and elevation angles are significant performance indicators for network planning, and QoS results. Figure 11 displays the RBF-NN layout in MATLAB. Figure 12 and Fig. 13 illustrate the predictions evolved from the proposed AI framework using Gaussian distribution, and MSE analysis, which also contains a performance evaluation of the optimized output.

Figure 12 depicts the Gaussian mixture distribution function of the RBF-NN using a shaded mesh plot in MATLAB. This distribution describes data that been clustered around a mean. The probability density is a plotted Gaussian bell curve with a peak at the mean, where each curve denotes a distinguished cluster with its own mean and covariance. This probability density can be calculated by k-means for each cluster’s mean and covariance, before either get updated at every iteration up to the maximum number of iterations, or no significant change in the Gaussian mixture distribution has occurred. The probability density converges to the outmost suitable clusters that meet optimization requirements. This is visualised as a bell curve with the peak as the evolved optimized value.

Figure 13 presents the MSE performance of the RBF-NN for all datasets that have been plotted on a logarithmic scale in MATLAB. Training MSE is declining as the number of iterations increase. The plot demonstrates the evolved optimized results against different values of ${\mathrm{h}}_{\mathrm{t}}$ that represent altitudes of a drone at various $\uptheta$. The optimized results scored the best MSE with the RBF-NN converging towards the best-fit value at around 129 iterations with the lowest MSE in comparison to non-optimized values. The performance indicator is fitting and reasonable as the final MSE is small, the NN converges with no overfitting occurring, before its best validation performance occurrence on the Gaussian mixture distribution. Therefore, the optimized parameters evolved are at an altitude of 21 m with an average improvement of -7dBm in RSS, which leads to an improved coverage distance D of up to 1.5 km, and an enhanced throughput T of 3.4 mb/s.

### Complexity analysis

We use complexity theory to analyse how the execution time of a simulation scales with inputs by considering the relationship between the number of basic operations with respect to the size of input, e.g., the number of iterations, and the number of drone altitudes and elevation angle. We use Big-O notation to mathematically express the complexity functions shown in Eqs. (38) and (39).

38 $$ \text{O}_{{\text{Ph}_{1} }} \left( \text{n} \right) = \text{n}^{2} + \text{C}_{{\text{Ph}_{1} }} $$

39 $$ \text{O}_{{\text{Ph}_{2} }} \left( \text{n} \right) = \text{n}^2 + \text{C}_{{\text{Ph}_{2} }} $$

where ${\text{O}}_{\text{ph}}\left(\text{n}\right)$ denotes the complexity level, n the number of operations, ${\text{C}}_{{\text{Ph}}_{1}}=0.70$, and ${\text{C}}_{{\text{Ph}}_{2}}=0.50$.

**Fig. 14:**
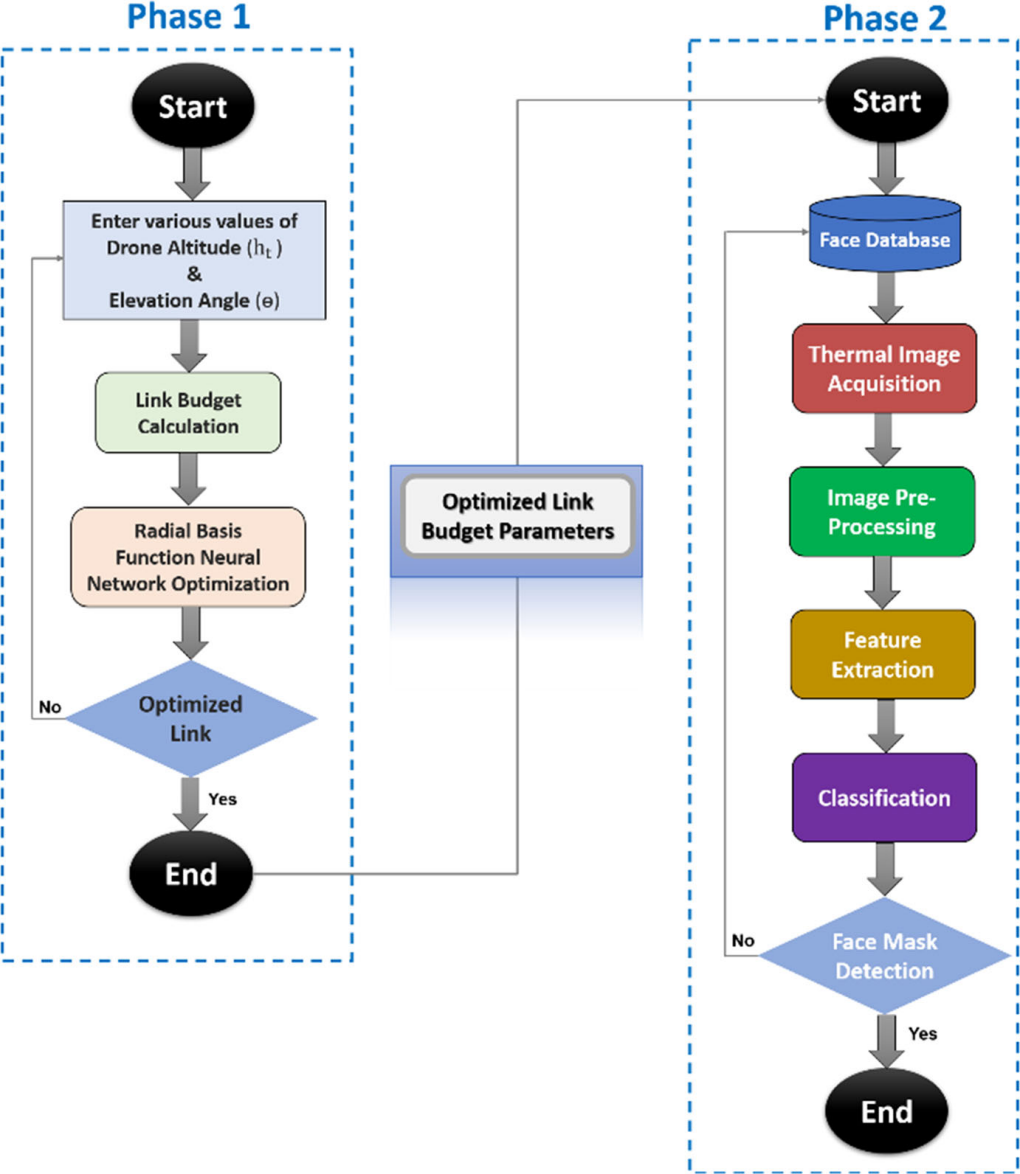
The AI framework complexity flowchart

**Fig. 15:**
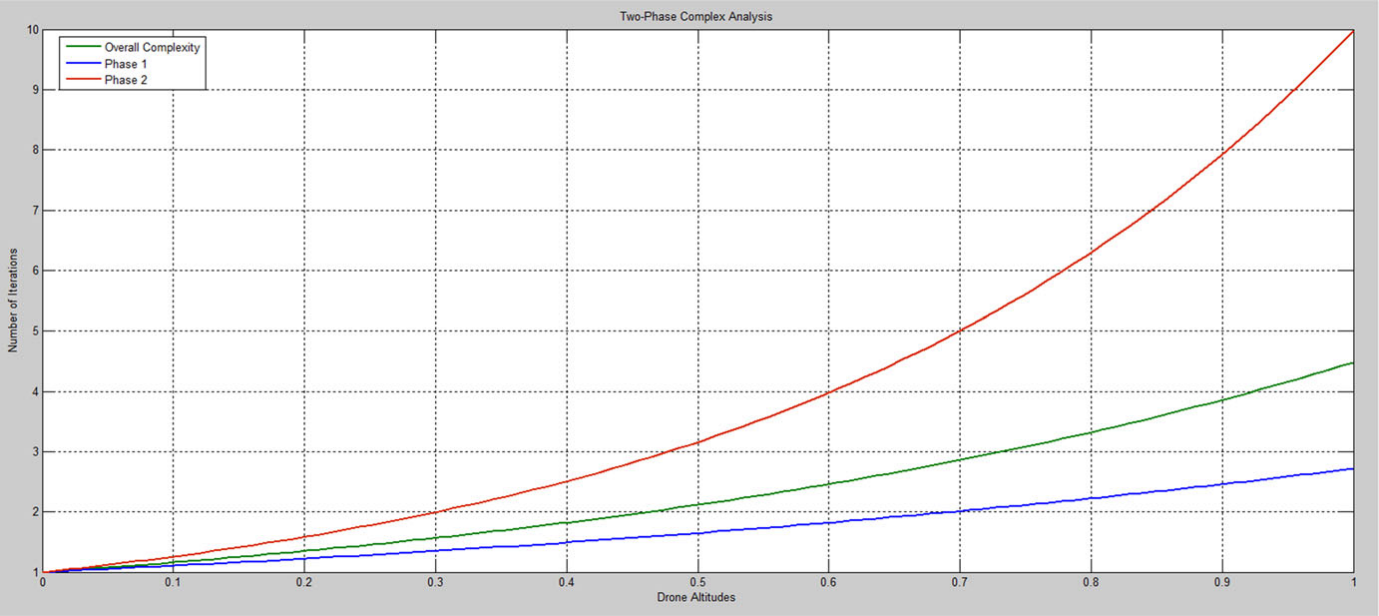
The AI framework complexity level

Figure 14 shows the level of complexity for the two phases of the proposed framework with the number of iterations rising exponentially in relation to the number of drone altitudes, as at each altitude the number of parameters considered are used as input to phase 2. While Fig. 15 reveals that whilst the overall time complexity rises in relation to the number of drone altitudes, the overall complexity of the proposed architecture is kept at a moderate level.

Working with drones is not without risks and anyone undertaking research that involves the use of drones will inevitably be confronted with a set of issues that are both hindering progress which often leads to sudden failure and the subject of ongoing research.

- Drone hovering flight stability. A sudden change in weather conditions, e.g., sudden gusts of wind, especially at low altitudes will affect both forward and hovering flight stability, especially hovering, which may throw the drone off course or cause a fall and crash to the ground. Correcting a drone’s reference direction, if it has remained in flight, requires additional gyroscopic measures and power consumption.
- Power consumption. A drone’s weight, fly time, connectivity and all on-board processing will determine its power consumption which is sourced entirely from on-board sources. Optimising power consumption requires migrating to other forms of energy or providing additional energy sources whilst maintaining a light weight as possible, transferring off-board as much of the on-board processing as possible, reducing the need for correcting a drone’s reference direction as much as possible to name just a few.

## Concluding discussion and future work

The integration of AI and Simulation is old wine [56] in new bottles [57]. In this era of the 4IR, smart cities contain smart things which can collaboratively enhance quality of life and people’s health and security. As part of the continuous effort to fight against the spread of coronavirus, this paper has shown that coupling a drone with AI may help with the fight against the pandemic. The proposed eye-in-the-sky drone system with its payloads of thermal imaging cameras and AI framework may help with detecting and recognizing persons wearing masks who are in violation of curfews and stay-at-home orders. The system can also perform basic diagnostic functions like elevated body temperatures to help with reducing the spread of infection. The AI framework evolves an optimized $\theta$ and ${\mathrm{h}}_{\mathrm{t}}$ to enhance connectivity, throughput, and power consumption in support. Results of the framework obtained using the MATLAB toolbox show promising output across three main findings: 82.63% accuracy with face mask detection; 0.98 F1-score which confirms that thermal imaging used for training, testing and validations is correctly classified; the Gaussian mixture distribution and MSE plots shows that optimized values offer reasonable channel performance with a mobile 5G antenna.

Drone technology is a promising robust and reliable wireless communication system because of its ability of rapid deployment and adoption of IoE and 5G technologies. We are currently investigating the potential use of an adaptive aerial system on-the-go for face mask detection for both indoors and outdoors settings e.g., airports, and shipping malls. Using a smart IoT application that can render a 3D urban environment on the go over Google Maps and track and trace multiple persons is increasingly being reported as future research and development work both in the academic literature as well as in industry. Several authors report ongoing research and development work in addressing drone hovering flight stability and energy consumption.
